# Genetic monogamy in high density populations of a threatened Mediterranean rodent

**DOI:** 10.1038/s41598-025-09003-0

**Published:** 2025-07-04

**Authors:** Ricardo Pita, José Jiménez, Joana Paupério, Benigno Cienfuegos, Alejandro Chamizo de Castro, Joshua Díaz-Caballero, João Queirós, Clara Ferreira, François Mougeot, Alfredo Anega, María Jesús Palacios, Juan José Luque-Larena

**Affiliations:** 1 MED – Instituto Mediterrânico para a Agricultura, Ambiente e Desenvolvimento, Évora, Portugal; CHANGE Instituto para as Alterações Globais e Sustentabilidade, Évora, Portugal; IFFA – Instituto de Investigação e Formação Avançada, Universidade de Évora, Évora, Portugal; Unidade de Biologia da Conservação, Universidade de Évora, Évora, Portugal; 2 Instituto de Investigación en Recursos Cinegéticos (IREC, CSIC-UCLM-JCCM), Ciudad Real, Spain; 3 CIBIO - Centro de Investigação Em Biodiversidade E Recursos Genéticos, InBIO Laboratório Associado, Universidade Do Porto, BIOPOLIS - Program in Genomics, Biodiversity and Land Planning, Vairão, Portugal; 4 FOTEX – Fomento de Técnicas Extremañas, Badajoz, Spain; 5 Dirección General de Sostenibilidad de La Junta de Extremadura, Mérida, Spain; 6 GPEX. Servicio de Conservación de La Naturaleza y Áreas Protegidas. Junta de Extremadura, Mérida, Spain; 7 Departamento de Biologia, Faculdade de Ciências, Universidade do Porto, Porto, Portugal; EBM – Estação Biológica de Mértola, Mértola, Portugal; 8 Departamento de Ciencias Agroforestales, ETSIIAA, Universidad de Valladolid, Palencia, Spain

**Keywords:** Cabrera vole, Genetic non-invasive sampling, Mating system, Population density, Spatial capture-recapture, Ecology, Evolution, Zoology

## Abstract

Monogamous mammal species often exhibit flexible mating strategies in response to socio-ecological factors such as population density, though the extent of this flexibility remains unclear. In this study, we investigated the genetic mating system of the Cabrera vole (*Microtus cabrerae*) in high density habitat patches from Extremadura (Spain), assessing whether it aligns with or deviates from the genetic monogamy previously found in low-density populations (< 20 individual/ha). Using genetic non-invasive sampling of vole faeces combined with spatial capture-recapture modelling, we first obtained precise estimates of population density. We then evaluated whether Cabrera voles display alternative mating tactics in these high-density patches by means of space use and parentage analysis. Results indicated that, even under unusually high population densities (> 90 individuals/ha), Cabrera voles exhibit genetic monogamous mating system, contrasting with the density-dependent variations often observed in other socially monogamous species. This suggests that Cabrera voles may have limited flexibility in their reproductive behaviours, potentially influenced by life-history traits such as strong pair-bonds, paternal care, and low levels of sperm competition, which likely promote paternity assurance and reduce the chances of extra-pair mating. These traits probably evolved in response to the limiting conditions that semi-arid Mediterranean environments impose to herbivores, influencing the most optimal mating strategies to the successful rearing of offspring. Overall, our findings highlight that not all monogamous mammal species exhibit flexibility in their mating strategies in response to population density. In the case of the Cabrera vole, being a near-threatened Iberian endemism, the predominance of genetic monogamy may increase its vulnerability to land-use and climate changes, given this mating strategy is generally associated with lower effective population sizes and overall genetic diversity. Conservation efforts for Cabrera voles should thus focus on preserving large and stable habitat patches, while improving landscape connectivity to mitigate potential population and genetic bottlenecks, and enhance the long-term viability of extant populations.

## Introduction

Mating systems describe the predominant reproductive strategy of species based on the number of mates that individuals of both sexes typically have in natural populations^1^. Most theories of the evolution of mating systems emphasize the importance of sexual behaviour as well as individuals’ space use, and the characteristics of pair bonds and parental care provided by each sex^2^. Monogamy represents a complex mating system in which breeding females generally occupy exclusive territories^3^ and neither sex can monopolize more than one mate, typically resulting in significant pair-bonding and biparental care^2,4^. From an evolutionary perspective, monogamous mating systems are remarkable, given that empirical studies and theoretical models typically associate the predominance of multiple mating as a proxy for lifetime reproductive success and fitness^5,6^. Understanding the factors affecting the emergence, variability and maintenance of monogamous mating systems is therefore an outstanding and still unresolved research challenge, yet essential for a comprehensive theory of mating systems^7^.

Among mammals, monogamy has long drawn considerable research attention due to its rarity (nearly 5%^4^), independent evolution^8^, and variability or intermittency in underlying social and reproductive behaviours^9,10^. Evidence suggests that, within monogamous mammal species (most common among rodents, canids, and primates, including humans), alternative reproductive tactics, or alternative behavioural phenotypes, conveying different reproductive payoffs may emerge in different contexts^10–13^. Such variation in the expression of mating behaviour is thought to be shaped by neurobiological processes involving specific socio-sexual and reward brain regions linked to pair-bonding circuits, which are modulated by genetic factors and external influences, thus enabling behavioural adaptation of monogamous mating systems to environmental pressures^14,15^. This has led to the differentiation between genetic monogamy, which involves association and exclusive mating with a single partner, and social monogamy, in which individuals also associate to a main partner but engage in multiple mating in and out of their social pair^16,17^. The predominance of each mating tactic within monogamous mammal species and populations thus emerges from individuals’ decisions reflecting their social and ecological environment^10,15^, and have been suggested to significantly impact population dynamics, dispersal, and gene flow^18,19^. These impacts may be further exacerbated in genetic terms when significant environmental change prompts typically monogamous species to transit towards non-monogamous mating systems in which male mate guarding and parental investment are presumably lower^10,20^. Assessing the drivers of variation in mating strategies among monogamous mammals and the potential for eventual transitions to other mating systems is therefore key to advance ecology, evolution, and conservation science^7,21^. However, the extent to which mating behaviour can adapt to varying social and ecological conditions across monogamous mammals remains uncertain. The relevance of socio-ecological environments modulating reproductive strategies in mammals remains poorly understood^22,23^, largely due to the challenge of surveying detailed demographic and parentage assignment data in natural populations, particularly for rare and elusive species^24–26^.

Population density has been identified as a main driver of individuals’ socio-ecological environment, and its effects on the amount of extra-pair mating have been well demonstrated among monogamous mammals^8,10,20^. Higher density typically increases the probability of social interactions and mating opportunities while also intensifying competition for mates, potentially enhancing the reproductive payoffs of extra-pair mating by increasing male reproductive success and allowing females to select mates and increase offspring genetic diversity^27,28^. This may increase the prevalence of social monogamy over genetic monogamy, or even the emergence of non-monogamous mating, such as polygyny. For instance, for prairie voles (*Microtus ochrogaster*), genetic monogamy is more prevalent during periods of low population density, while at high population density, male territories may overlap with those of multiple females, potentially increasing extra-pair matings and paternities, hence reducing genetic monogamy^29–31^. Occasionally, some prairie voles may also exploit polygyny mating alternatives in environments offering increased access to multiple mates^10,32^. This variation in prairie vole mating strategies emerges from individuals choice to adopt a monogamous tactic (known as ‘resident’), in which individuals defend small home ranges that closely overlap with their mating pair and minimally with other adult conspecifics, or a non-monogamous tactic (known as ‘wandering’), in which males typically occupy larger home ranges that overlap with multiple females and males, without establishing pair bonds^10^. Other examples of monogamous mammals showing higher rates of extra-pair paternity in densely distributed populations include the alpine marmot (*Marmota marmota*)^27^, the American beaver (*Castor canadensis*)^33^, the Tome’s spiny rat (*Proechimys semispinosus*)^34^, and the Brazilian guinea pig (*Cavia aperea*)^35^. These transitions make it unclear whether genetic monogamy occurs as a consequence of the life history traits of socially monogamous species, or is mainly driven by changes in the socio-ecological environment due to population density^17,36^. Therefore, studies assessing how mating strategies vary with population density in socially monogamous mammals are crucial to improve our understanding of the development, maintenance and variation of monogamy, and on how natural populations will be able to adapt and persist in rapidly changing environments, particularly among species of conservation concern^37^.

Here we address this issue focussing on the monogamous Cabrera vole (*Microtus cabrerae*)^25,38–40^. The Cabrera vole is a near-threatened Iberian endemism with global declining trends^41^, being largely restricted to small habitat patches of herbaceous wetland providing food, thermal refuge, and protection from predators^42–44^. Within habitat patches the Cabrera vole typically occurs at low densities, rarely exceeding 50 individuals/ha and often remaining below ~ 20 individuals/ha^26,45–47^, being organized in small family groups consisting of a bonded breeding pair and their offspring. Bonded breeding pairs often share a common range, with limited overlap with home ranges of adjacent family groups^39,44,48^. Such socio-spatial organization, together with the evidence of biparental care^38^, the reduced sexual size dimorphism^49^, the trends toward even sex ratios of populations^45,50^, and the lack of evidence for extra-pair paternity^25^ suggests that, at low densities, the Cabrera vole is genetically monogamous. However, the species may occasionally reach high densities (above ~ 100 individuals/ha)^51^, though such occurrences appear relatively uncommon and localized, likely triggered by particularly favourable conditions and high reproductive output, rather than reflecting periodic abundance fluctuations as documented in other vole species e.g^52,53^. Despite uncommon, such high-density populations may still have important ecological implications, potentially altering social structures and mating strategies, thus offering an excellent opportunity to test variations under natural conditions. In addition, in some local populations the sex-ratio may be biased towards females^45^, while males may also show a slight tendency for larger-home ranges and overall activity than females^39,43,45^. Together, these observations suggest that under certain conditions there may be some deviation to the typical genetic monogamous mating found in Cabrera vole populations (e.g. towards social monogamy or polygyny), with population density potentially driving eventual changes in individual mating behaviours^10^.

Based on genetic non-invasive sampling (gNIS) of vole faeces in a priori high density habitat patches, we used spatial capture-recapture (SCR) modelling to precisely estimate male and female population density and space use, and parentage analysis to test the hypothesis that, similarly to other monogamous voles^11^, the Cabrera vole may adopt alternative mating tactics when density is high and individuals have access to multiple potential mates. Specifically, we evaluated the prediction that extra-pair mating should be present in high-density patches, in contrast to its absence previously verified in low-density patches^25,46^. The emerging genetic mating strategy in high-density patches should therefore be more consistent with social monogamous or possibly polygynous mating systems, both of which involving multiple paternity. This should also result in relatively high spatial aggregation among females, together with relatively high spatial evenness between sexes^11^. If correct, our hypothesis would provide support for the ability of Cabrera voles to shift from genetic monogamy at low population density, to social monogamy with eventual polygyny at high population density. Conversely, genetic monogamy may represent a relatively stable life-history trait of the species, which remains largely invariable to changes in density and associated socio-ecological context^17,36^.

## Methods

### Study area and sites

The study was carried out in Cáceres Province, Extremadura region (western Spain), which is characterized by a Mediterranean climate (mean annual rainfall of 491 mm, concentrated in November–April, and mean annual temperature of 16.1 °C, ranging between 8 °C in January and 29 °C in July^54^. Geo-morphologically the area is characterized by flat or gently rolling lowlands with slopes from 0 to 9°. Land cover is dominated by a multifunctional agro-silvopastoral system, the ‘dehesa’ or ‘montado’, as well as by transitional woodlands and shrublands, together with open grasslands and old fields (pseudo-steppes). Land-use focuses mostly on extensive livestock raising of sheep, cattle, pigs, and goats. Oats, wheat, barley, and rye are cultivated in long rotation cycles in parts of the ‘dehesas’. Within these agricultural land mosaics, suitable habitats for Cabrera voles are scattered along uncultivated marginal habitats, often associated to small seasonally drying streams dominated by wet herbaceous cover, usually dense, and interspersed with or bordered by some shrubs.

**Fig. 1:**
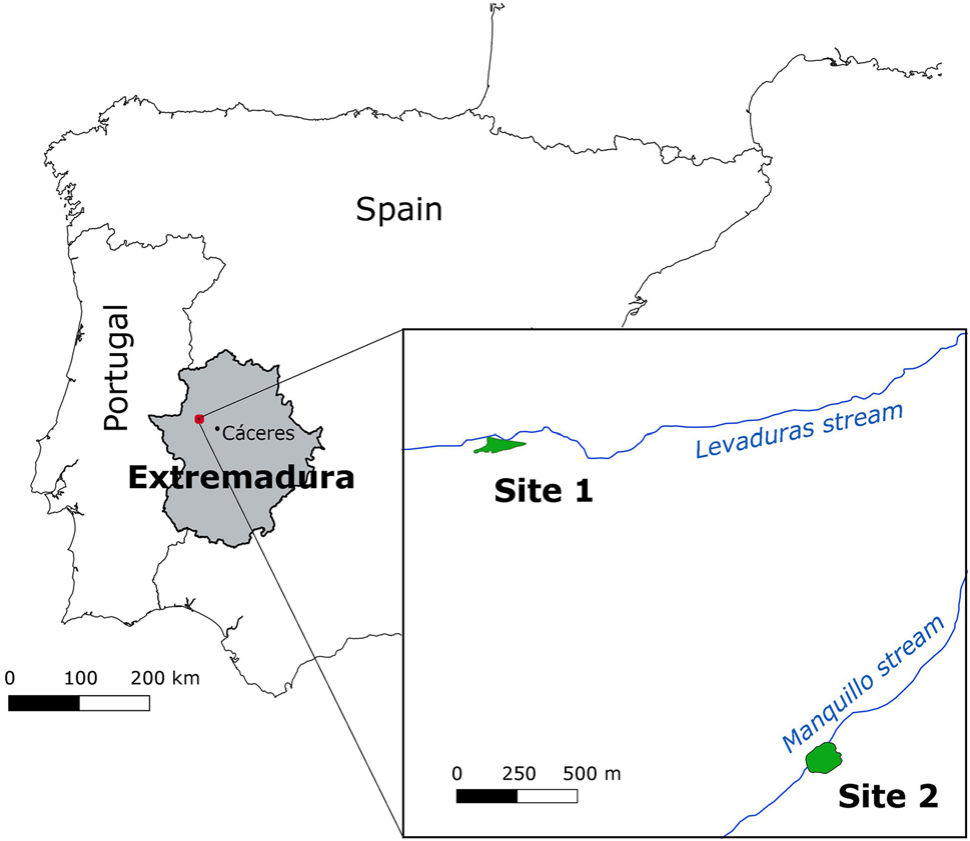
Location of the two selected habitat patches in Extremadura (Site-1 and Site-2) selected for the study.

In the context of a larger research program towards the assessment of the conservation status of the species in Extremadura (project FEDER—POCTEP—0068_REDTI_4_E, Taejo Internacional Rede), field work was conducted in spring 2021, when breeding activity of Cabrera voles is expected to peak^44^. Two relatively large habitat patches, hereafter Site-1 (0.66 ha) and Site-2 (1.38 ha), located ca. 30 km northwest of Caceres city (Brozas municipality) were identified as potentially holding high local population densities of Cabrera voles, based on the large amount of presence signs found at these sites^47^ (see Fig. 1). The two habitat patches were separated by 1.8 km, with Site-1 bordering the eastern side of the Brozas lake, which dams the Levaduras stream, and Site-2 being associated to a fenced grazing exclosure in a shallow soil depression crossed by the Manquillo stream (see Fig. 1).

### Genetic non-invasive sampling of voles

Cabrera vole surveys in each patch were based on throughout searches of the species-specific signs (e.g. tunnels of 4–6 cm diameter made on grasses, accumulations of grass clippings, latrines with faeces of 5–9 mm length and 2–3 mm width, see^47,55^). Cabrera vole signs are easily recognizable where other species producing potentially confoundable signs (e.g. *M. arvalis*, *M. agrestis*) are absent, as is the case of our study area^56^. Searches were performed along zigzag transects guided by continuous tracking and inspection of microhabitats suitable for the species within the whole habitat patch, following the procedures described in^47^. Given the relatively large size of selected patches, this required a total of 3 visits in Patch 1 (between 2^nd^ and 17^th^ June 2021) and 4 visits in Patch 2 (between 24^th^ May and 16^th^ June 2021), involving 2–3 trained observers simultaneously searching different parts of the patches to ensure thorough coverage of their entire surface. These time intervals were considered short enough to assume negligible losses (deaths and emigration) and gains (births and immigration), such that each local populations could be considered as closed for vole density estimation (see below). During surveys, genetic non-invasive sampling of vole faeces was performed for individual identification^25,46^ by collecting faeces from latrines that were at a minimum distance of 0.5 m from any other latrine already sampled^26^. The location of each faecal sample collected was recorded with precision < 0.5 m, using a Topcon FC-6000 GPS tracker (Topcon Corp., Japan). Each sample consisted of up to 12 of the freshest faeces found in each vole latrine (usually on top) to ensure they had been deposited within the last few days and were suitable for genotyping^25,26,46^. Samples were collected from each latrine using sterilized tweezers and were kept in labelled individual 2 mL microtubes containing 96% alcohol, and stored at −20 ºC until DNA extraction.

### Molecular analyses

DNA was extracted from faecal samples using the E.Z.N.A.® Tissue DNA Kit (OMEGA bio-tek, GA, USA) following the manufacturer’s instructions, with an initial digestion step using a lysis washing buffer^57^ for 15 min at 56ºC^47^. Samples were genotyped for a set of 11 specific, highly polymorphic microsatellites: Mc18 (accession number MH264524)^25^, Mc24 (accession number MH264526)^25^, Mc30 (accession number MH264528)^25^, Mc02 (accession number MH264520)^25^, MSMM-3 (accession number AB016154)^58^, Mar-03 (accession number EF666981)^59^, Mar-16 (accession number EF666983)^59^, Mar-76 (accession number EF666987)^59^, MAG25 (accession number EF409379)^60^, Ma25 (accession number EF177204)^61^, and Mc07 (accession number MH264521)^25^, which is potentially located on the X chromosome. These loci have been previously validated for accurate individual identification, with estimated probabilities of identity among unrelated and related individuals of 3.3 × 10^–12^ and 4.2 × 10^–5^, and probability of exclusion of 0.999^25^. Species ID was confirmed for a subset of genotyped samples (ca. 60%, see Results) using a small fragment of mitochondrial DNA, D-loop^46,62^. The samples were also sexed using two small-sized sex chromosome introns (DBX5-S and DBY7-S^25^). To account for genotyping errors (e.g. allele dropout and false alleles) and to obtain a consensus genotype, each multiplex reaction was replicated four times (three times for the sex chromosome introns amplification). PCR reactions were performed in a final volume of 10 μL, consisting of 4 μL of Qiagen© Multiplex PCR Kit Master Mix, 1μL of DNA, and primer concentrations and thermal profiles according to previous works^25,26,46,47^. All products were sequenced on an ABI3130 Capillary Sequencer (Applied Biosystems). The extractions and PCR reactions of the non-invasive samples were performed in physically isolated rooms, and all the equipment used was sterilized with bleach and ethanol and exposed to UV light before and after usage. Aerosol-resistant pipette tips were used, and negative controls were included in each manipulation, maintaining conditions to monitor and reduce risk of DNA contamination^63–65^. Allele calling of the microsatellite loci and sex chromosome introns were performed using GeneMapper (v.4.0; Applied Biosystems), while D-loop sequences were analysed with Geneious (v.8.0^66^). Consensus genotypes for each sample were obtained by analysing all replicate genotypes with the software Gimlet (v.1.3.3^67^). For genotypes differing by up to two loci or with up to two missing data, additional PCR replicas were performed, to try to complete the genotypes, and check for genotyping errors. Genotyping error rates were estimated using the software Pedant^68^, with 10,000 search steps. Since the software only allows the comparison of two replicates, all possible pairwise comparisons were performed, and the results were averaged. Sample consensus genotypes were then compared with each other to identify individuals. The criteria used to assign samples to individuals was very strict, with only individuals differing in more than two loci assigned as new captures^47^. The number of alleles (Na), expected heterozygosity (H_E_), observed heterozygosity (H_O_) and overall inbreeding (F_IS_) were calculated for all loci except the Mc07, using ‘adegenet’^69^ and ‘pegas’^70^ in R version 4.3.0^71^.

### Spatial density modelling

Vole density was estimated in each patch using spatial capture-recapture (SCR) modelling^72–74^, thereby allowing heterogeneous detection probabilities due to uneven spatial distribution of animals relative to sampling locations, and spatial variation in animal densities due to eventual variation in habitat features within surveyed patches^75,76^. Briefly, SCR models are hierarchical models composed of a submodel for the detection of individuals, conditional to their location, and a submodel for the distribution of individuals in space (i.e., density, *D*). These models assume that animals move around a central point referred to as activity centre (*AC*), which is inferred from the different detection sites of recaptured individuals rather than the coordinates of detection events^77,78^. Density is then modelled as a non-homogeneous point-process distribution of latent *AC*s over the state space *S*^77^. SCR models also assume that detection probability of each individual decreases with the distance between its *AC* and sampling locations, according to a detection function describing animals’ movement. Here we considered the most common detection function, the half-normal, which is specified by (i) the scale parameter (*σ*) describing how fast the detection probability decreases with increasing distance to the individuals’ *AC*s (often used to indicate home-range size); and (ii) the baseline detection probability ${(\lambda }_{0}$) which describes the probability of detecting an individual at its *AC*^74,77^.

SCR modelling was implemented by discretizing space at each selected patch with a grid of hexagonal polygons with 10m^2^, with gNIS-based detection sites corresponding to the centroids of the resulting polygonal grids (polygon detectors) to create a uniformly spaced sampling grid. Hexagons have been shown to be appropriate in SCR approaches relative to square polygons, reducing the sampling bias associated with edge effects, given their low proportion between perimeter and area^79^. The chosen polygon size was based on preliminary analysis testing the relationship between the scale parameter (*σ*) of the half-normal detection function and the distance between polygon centroids, which should be lower than 1.5 times *σ*^80^, while balancing computational efficiency.

As regards to the submodel of density, we included the linear and quadratic effects of altitude (extracted from the Spanish National Centre for Geographic Information, CNIG, at 2 × 2m^2^ resolution), assuming that this variable should reflect important habitat characteristics for voles, particularly regarding inundation *versus* non-inundation locations. We assume the activity centres ${s}_{i}$ were non-homogeneously distributed in the space according to an intensity function $\lambda \left(s\right)$.

$$\lambda \left(s\right)=\text{exp}({\beta }_{1}\cdot Elev+{\beta }_{2}\cdot {Elev}^{2})$$

$${s}_{i}\propto \lambda \left(s\right)$$

$${z}_{i}\sim Bern(\psi )$$

where ${z}_{i}$ is a partially latent binary indicator variable that describes the membership of individual *i* in the population, and $\psi$ the parameter of the $M$ data augmentation.

Assuming that encounter frequencies are Poisson-distributed and a decreasing function of the Euclidean distance $\parallel {s}_{i}-{x}_{j}\parallel$ between individual activity centre ${s}_{i}$ and trap location ${x}_{j}$, the expected encounter rate can be specified as:

$${\lambda }_{ij}={\lambda }_{0}\text{exp}(-\frac{{\parallel {s}_{i}-{x}_{j}\parallel }^{2}}{2{\sigma }^{2}})$$

The likelihood for the true encounter frequencies is:

$${y}_{ijk} | {z}_{i }\sim Poisson({z}_{i}\cdot {\lambda }_{ij})$$

Population size is derived from the sum of indicators:

$$N=\sum_{i=1}^{M}{z}_{i}$$

Selected habitat patches were treated as strata, each with a population size parameter (*N*), combined in a single, unified multi-strata closed model for purposes of improved estimation of parameters that can be shared between the two patches, while accounting for possible variation (patch-specific) in population density (*D*)^81^. In particular, in order to improve parameter estimates due to reduced number of spatial recaptures (see Results), we implemented our model with shared scale parameter (*σ*) across selected patches^82–85^. In addition, because the incorporation of detections of unidentified individuals is referred to result in more precise information than standard SCR models^86^, we implemented the random thinning SCR modelling approach described in^87^. This approach incorporates encounters of both known and unknown identity gNIS samples using a natural mechanistic dependence between samples arising from a single observation model^87^. Encounter observation histories for *N* observed individuals in a given strata were assumed to arise from a Poisson distribution. The true encounter frequencies for the *n* individuals with at least one detection are what would be observed if all samples were individually identifiable. The number of unobserved individuals with “all-zero” detection histories (*M-n*) was estimated using the data augmentation within a Bayesian framework, choosing a value such that *M* ≫ *N*^86^, which, based on preliminary analysis, was set as *M* = 300 potential individuals.

We included the sex and the linear and quadratic terms of the sampling effort (transect length within each polygon) as fixed-effect covariates of the baseline detection rate ${(\lambda }_{0}$), with the intercept fixed as 0 to account for polygons not surveyed. This allowed to account for potential differences in detection probability between sexes. We also included sex as a fixed-effect covariate of *σ*, a parameter shown to differ between sexes in another vole species^74^*.* To accelerate the sampling MCMC process we use a habitat raster converted to a matrix of 1 s and 0 s, with 1 indicating suitable habitat (limit of each patch area) using the package *makeJAGSmask*^88^.

Posterior probabilities of model parameters were calculated using 3 independent MCMC chains, with 50,000 iterations each, and a burn-in of 1,000 iterations, using NIMBLE version 0.13.1^89^ in R version 4.3.0^71^. Finally, we assessed MCMC model convergence and mixing by visually inspecting trace plots and the Gelman-Rubin statistics R-hat^90^, estimated using the R package *coda*^91^.

### Mating strategy inferences

Kinship relations were assessed using the genetic parentage analysis software COLONY 2.0.6.8^92^. This software uses information from multilocus dominant or codominant markers (including null alleles), or a mixture of both type of markers, to infer full-sibship and parentage. Unlike other commonly used approaches, COLONY infers parentage and sibship jointly, and full-pedigree likelihood is considered over the entire pedigree using multilocus genotype data, rather than for pairs of individuals. Four possible mating systems were considered: 1) monogamous mating for both sexes; 2) polygamy for both sexes (promiscuity); 3) polygyny; and 4) polyandry. We input the genotyping error rates given by the Pedant analyses. Given the likelihood that the locus Mc07 is on the X chromosome, we removed this locus from parentage analyses but used the Mc07 genotype information to exclude potential paternities and maternities before running COLONY to improve performance of the software. We did not include inbreeding rates in our analyses. We used a default value of 0.5 for the proportion of sampled parents^92^, as preliminary analyses showed no differences in the results^25^. All individuals from the two sites were included in the same dataset. To aid in kinship analysis, we compared the D-loop haplotypes among individuals and used that information for excluding maternity and sibship in the input for COLONY. To check the reliability of the results, we run six replicates of “very long” runs for each of the four different mating system assumptions, with different random numbers of seeds. The resulting full-sibling and half-sibling (poly-systems) assignments were only considered if these were consistently recovered in four of the six runs (*i.e.*, the six replicates of each of the four types of mating systems) with a probability > 0.95.

In addition to the genetic mating system, we also estimated spatial segregation indices^93^ between males and females to describe sex-specific socio-spatial organizations in each site. Specifically, we measured two dimensions of sexual segregation, namely spatial exposure (or spatial isolation) and spatial evenness (or spatial clustering). Spatial exposure refers to the extent that members of one group encounter members of another group (or their own group, in the case of spatial isolation) in their local spatial environments. Spatial exposure ranges from 0 to 1, where values closer to 0 indicate minimal interaction between groups (high isolation) and values closer to 1 suggest frequent encounters (low isolation). Spatial evenness, or clustering, refers to the extent to which groups are similarly distributed in space. The most widely used measure of evenness is the dissimilarity index. Conceptually, dissimilarity measures the percentage of a group’s population that would have to move in order to create a uniform distribution of the population. also ranges from 0 to 1, with values closer to 0 indicating a uniform distribution (complete integration) and values closer to 1 reflecting pronounced spatial clustering (complete segregation)^93^. While these segregation measures have been developed in the geographical and sociological literature, they may be used in ecological segregation research and help understanding socio-spatial organization and infer mating tactics in animal species. Although many factors (e.g. resource distribution, habitat selection) may affect the spatial distribution of voles within patches, typical monogamous mating strategies are expected to result in relatively moderate to high dissimilarity, and gender-specific exposure patterns due to stable pair bonds and territoriality, whereas non-monogamous systems are expected to show lower dissimilarity and higher interaction or exposure among and between sexes due to less rigid social and spatial structures. Estimates of dissimilarity and exposure indices in each selected patch were calculated using the R package *seg* version 0.5–7, using the *spseg* function, which assumes a negative exponential distance decay function for modelling the effect of the distance on group interactions^94^.

## Results

### Genotyping and genetic patterns

**Fig. 2:**
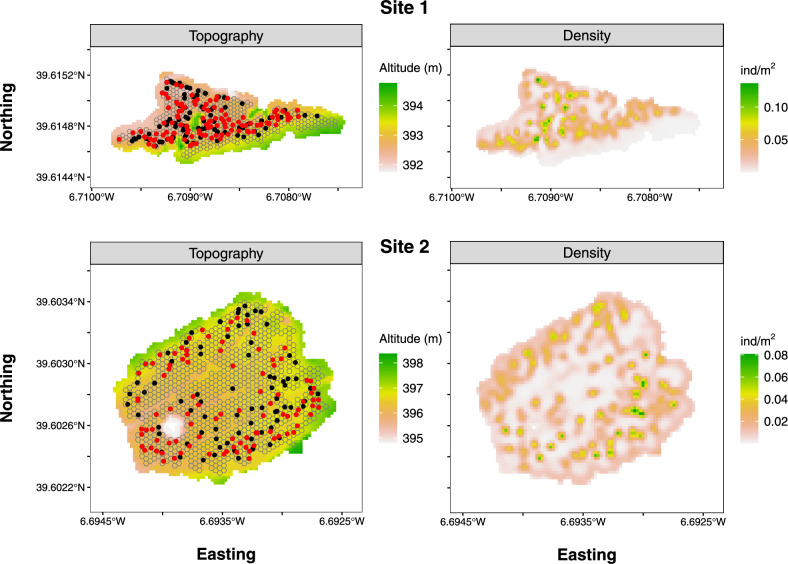
Maps on the left – Distribution of Cabrera vole faecal samples collected in each study site, with black dots indicating those that could not be genotyped and red dots indicating those that were successfully genotyped (see main text). Background gradients represent the variations in altitude (in meters). The grid of hexagonal polygons with 10m^2^ used in SCR modelling is also shown. Maps on the right – Predicted distribution and mean density of activity centres of Cabrera voles in each study site, estimated using spatial capture-recapture (SCR) modelling**,** based on the spatial detection histories of genotyped individuals. Created using R version 4.3.0 (https://www.r-project.org).

A total of 199 and 173 faecal samples were collected from Site-1 and Site-2, with successful genotyping of 82 and 72 samples, respectively (Fig. 2; Supplementary information 1 for full genotyping data), resulting in an overall genotyping success of 41.4%. Error rates were low across the 11 loci among the total of 154 genotyped samples (mean ± SD dropout rate = 0.92 ± 0.04%, false alleles rate = 0.02 ± 0.00%, see Table S1 in Supplementary information 2). The D-loop sequencing on a total of 93 samples confirmed the species identification in all, and revealed only two haplotypes of the D-loop marker (Hap1 and Hap7, GenBank accession numbers FR695397 and FR695403, respectively). Hap1 was observed in 83 samples, while Hap7 was observed in 10 samples. In one sample from Site-1, it was not possible to differentiate between the two haplotypes. The Na per locus ranged from 5 to 12 (mean ± se: 7.7 ± 0.71), H_E_ varied between 0.38–0.85 (mean ± se: 0.67 ± 0.08), and H_O_ varied between 0.14–0.88 (mean ± se: 0.69 ± 0.05). The overall F_IS_ value was 0.064 (see Table S2 in Supplementary information 2). Overall, a total of 131 individuals were identified, with 63 in Site-1 (38 females and 25 males), and 68 in Site-2 (42 females and 26 males). This corresponded to about 15% of the total genotyped samples being ‘recaptures’, with a mean ± se of 2.3 ± 0.1 capture times per recaptured individual.

### Density estimates

The SCR modelling confirmed high population densities in both study sites (139 and 91 individuals per ha in site 1 and 2, respectively). Mean and respective 95% Bayesian credible interval (95%BCI) of posterior estimates of absolute population size tended to be higher in Site-2 than in Site-1, though with no clear evidence for differences between them (Table 1). However, despite the notably high values obtained in each site, the 95%CI for the mean posterior density estimates were apparently higher in Site-1 (Table 1). The mean posterior estimates of *σ* and ${\lambda }_{0}$ did not differ between sexes (Table 1). The distribution of individual activity centres, which are probabilistic predictions obtained from a fitted SCR model^78^, were fairly uniform in both sites (Fig. 2), though the small scale variations in altitude within each site apparently affected vole density, with intermediate altitudes associated with higher densities, particularly in Site-1 (Table 1 and Fig. S1 in Supplementary information 2). The model also confirmed a female-biased sex ratio in both sites (Table 1).

**Table 1:** Resume of posterior estimates (mean and 95% Bayesian credible intervals, BCI) of the multi-strata SCR model parameters used to estimate Cabrera vole density for each study site.

**SCR Parameter**	**Site 1**		**Site 2**	
	**Mean**	**95% BCI**	**Mean**	**95% BCI**
Population size (N)	119.02	96.00–141.00	136.06	112.78–165.48
Realized number of Males	40.67	30.00–51.00	41.4	30.00–54.00
Realized number of Females	78.35	56.00–100.00	94.67	69.00–123.00
Density (D, individuals/ha)	139.83	112.78–165.65	91.47	72.60–110.92
Sex ratio (probability of an individual being a male)	0.34	0.25–0.44	0.31	0.21–0.40
Data augmentation parameter (psi)	0.59	0.47–0.74	0.68	0.54–0.86
Males *σ*	2.75	2.38–3.14	2.75	2.38–3.14
Females *σ*	2.18	1.91–2.45	2.18	1.91–2.45
Sex as covariate of ${\lambda }_{0}$ (females term)	0.01	−0.26 – 0.28	−0.02	−0.29 – 0.26
Sampling effort as covariate of ${\lambda }_{0}$ (linear term)	0.39	0.20–0.58	0.59	0.38–0.79
Sampling effort as covariate of ${\lambda }_{0}$ (quadratic term)	−0.18	−0.30 – 0.07	−0.20	−0.29 – 0.10
Altitude as covariate of density (linear term)	−0.86	−1.63 – −0.17	0.24	−0.13 – 0.61
Altitude as covariate of density (quadratic term)	−1.3	−2.23 – −0.42	−0.18	−0.55 – 0.15

### Genetic and social mating system

**Fig. 3:**
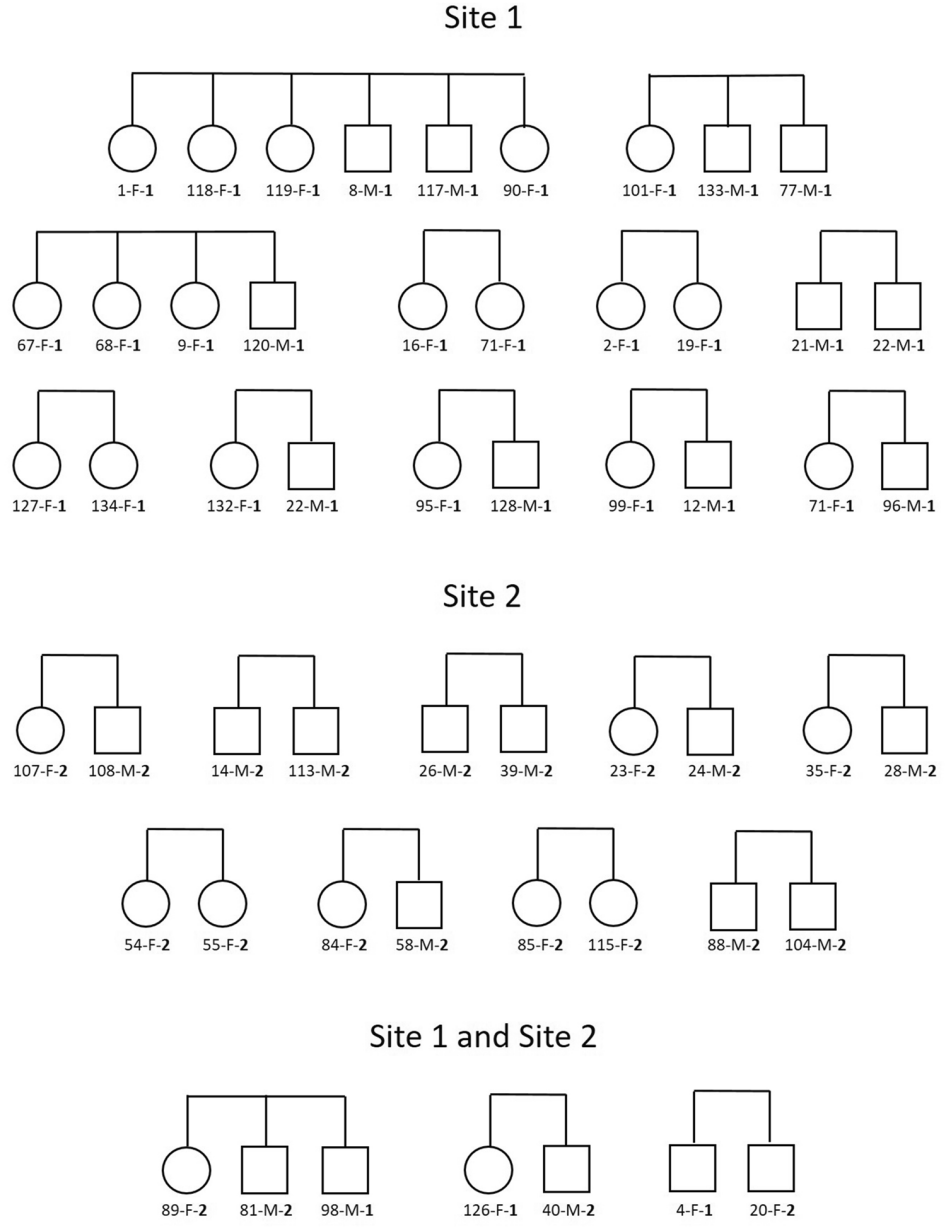
Full-sib relationships identified by COLONY assuming genetic monogamy in each study site. Circles: females, squares: males. Each vole is coded with an individual number, a letter indicating its sex (M – males; F – females), and a digit (1 or 2, in bold) corresponding to the site where it was ‘captured’.

When assuming a monogamous mating system, the identification accuracy of full‐siblings in COLONY was far better than when assuming non-monogamy. In addition, no half-siblings were found under non-monogamous mating systems, which would be indicative of multiple paternity. Thus these results support a genetic monogamous mating system of Cabrera voles in both study sites. Specifically, under genetic monogamy, COLONY correctly identified a total of 23 full-sib relationships among 54 individuals from both study sites, with 11 full-sibling pairs identified in Site-1, 9 in Site-2, and 3 between individuals from the two sites, which is indicative of voles dispersal (at least 3–4 individual movements) from different family groups (Fig. 3). By contrast, only 6 full-sibling pairs identified when assuming promiscuous mating (all in Site 1), and 8 pairs (7 in Site-1, and 1 in Site-2) when assuming polygyny or when assuming polyandry (Table S3, Supplementary information 2).

**Fig. 4:**
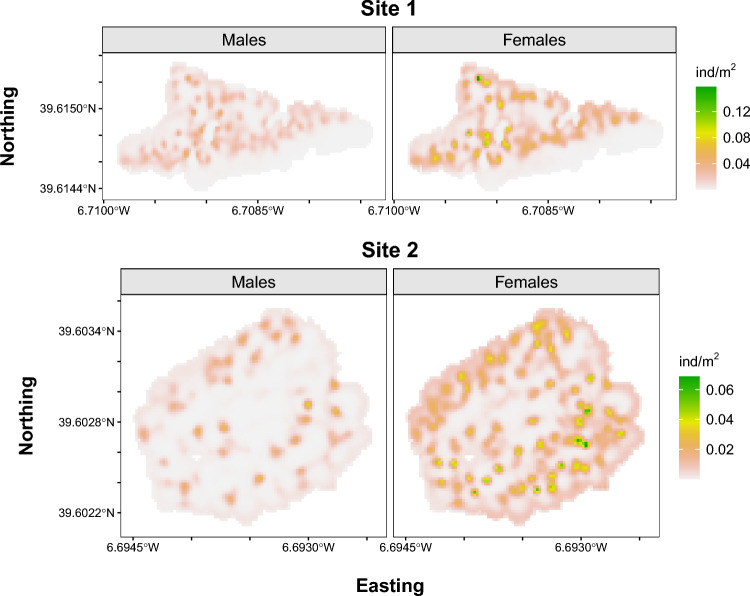
SCR-based mean density surfaces of activity centres of Cabrera vole males and females in each study site. Created using R version 4.3.0 (https://www.r-project.org).

Regarding socio-spatial organization, the dissimilarity index between males and females were 0.40 in Site-1 and 0.49 in Site-2, indicating moderate levels of sexual spatial segregation in both study sites (see Fig. 4). The exposure index of females to other females were notably high (0.74 in Site 1 and 0.80 in Site 2), suggesting significant interaction or proximity among females. By contrast, the exposure index of males to other males were comparatively lower in both sites (0.50 in Site-1, and 0.53 in Site-2), indicating their tendency to maintain territorial boundaries with fewer direct contact with other males. Additionally, females exhibited an exposure index to males of 0.20 in Site-1 and 0.26 in Site-2, while males had an exposure index to females of 0.47 and 0.5, respectively. These patterns suggest that males may frequently interact with females within their territories, while females’ exposure to males is less frequent and potentially more selective.

## Discussion

Many genetically monogamous mammal species may adopt alternative mating tactics in response to changes in their socio-ecological environment, mostly driven by variations in population density^8,20^. The extension to which flexibility in mating tactics occurs among genetically monogamous mammals is however uncertain, making it difficult to predict the adaptive potential and reproductive success of such species in spatially and temporally heterogeneous environments^17,36^. In our study focused on the near-threatened Iberian endemic Cabrera vole, direct parentage analysis revealed that genetic monogamy prevailed even under unusually high densities (> 90 individuals/ha), consistent with patterns previously observed at lower densities (< 20 individuals/ha)^25,26,46^. This inference aligns with the sex-based spatial structuring observed within the study populations, which, despite focused on population-level patterns, is consistent with male–female pairings occurring at finer, individual-level spatial scales^39^. The moderate to high levels of heterozygosity and low overall inbreeding coefficient also supported genetic monogamy, aligning with patterns reported in lower-density populations^25,26,46,47^, and in other monogamous mammals under demographic connectivity or long-term pair bonding limiting inbreeding^95–97^. Therefore, contrary to many monogamous mammals presenting density-dependent intraspecific variation in the extent to which genetic monogamy occurs^10,34^, the Cabrera vole seems to have relatively low plasticity in its reproductive behaviour across variable population densities. While other factors may influence eventual variation in species mating tactics (e.g. habitat quality^98^), our study suggests that genetic monogamy in the Cabrera vole seems to be a consequence of particular life-history traits and/or phenotypic constrains, independent of population density. This finding has conservation implications, suggesting a limited adaptive potential of this threatened species to changing socio-environmental conditions, making it potentially more vulnerable to environmental fluctuations, habitat alterations and climate change^5^.

### What drives the maintenance of genetic monogamy in Cabrera voles?

Several key life-history traits may explain the lack of extra-pair mating and the emergence of genetic monogamy of Cabrera voles even at high population densities, including those related to social structure, breeding seasonality and synchrony, or parental care^5,17^. In particular, the close association between a male and a female typically observed in Cabrera voles^38,39^, makes it easier to mate guard and harder to engage in multiple mating involving increased energetic demands and predation risk^17,99^. In addition, because investment in reproduction typically peaks during early spring^44^, some level of synchrony in females’ receptivity might also contribute to limit extra-pair paternity as males become too busy guarding their mate to engage in multiple mating^100^. Also, the benefits of paternity assurance and parental care typically provided by males in addition to that provided by females^38,44^may further contribute to limit extra-pair mating, thus promoting genetic monogamy independently of local density. In addition, infanticide by unrelated males is known to occur across mammalian taxa, and monogamy has been proposed as a counterstrategy to reduce this risk e.g. ^101^. In the Cabrera vole, where males are closely associated with their mates and contribute to offspring care, genetic monogamy can similarly function to reduce the risk of infanticide^12^. This suggests that genetic monogamy in the Cabrera vole may have primarily evolved because selection favoured males that were more affiliative toward females and that provided parental care only when paternity was certain, thereby avoiding investment in offspring from other males^17^. Such distinctive selection may have been driven by the limiting environmental conditions characteristic of semi-arid Mediterranean regions, where green herbaceous vegetation is scarce and often absent during the dry summer periods^44,49^, thus imposing environmental pressures that favour cooperative strategies among sexes ensuring the successful rearing of offspring. Therefore, like other rodents native to semi-arid environments also documented as truly monogamous (both socially and genetically) (e.g. the California mice, *Peromyscus californicus*^102^), maintaining strong pair bonds, providing paternal care, and remaining genetically monogamous should provide greater reproductive success for Cabrera voles than otherwise, even under unusually high vole density potentially increasing the opportunities for extra-pair mating. Notably, this system may apparently operate alongside a female-biased sex-ratio, a pattern occasionally also found in lower-density patches^39,45^. However, even though our sampling method was designed to ensure complete coverage of the surveyed habitat patches, we cannot rule out the possibility that some subdominant males lacking well-defined home ranges could be less effective at marking territories, potentially leading to their droppings going undetected in our surveys. On the other hand, the estimated larger number of females suggests that those that remain unpaired might have some role in raising offspring of breeding relatives (kin selection), thus contributing to the care and protection of the group’s offspring^8^. Kin-based female groups are widespread among mammals^103^, and such structure is theoretically expected in systems where spatial proximity increases the likelihood of interacting with close relatives^104^. This is supported by the spatial overlap analysis, which unveiled a relatively high exposure of females to other females, together with a high spatial segregation among males, a pattern that has been also observed in low density patches^39^, and that is commonly found among many vole species^8^. Although more research is needed to confirm the occurrence of cooperative breeding in the Cabrera vole, this trait has been shown to be largely restricted to genetically monogamous mating with limited extra-pair mating^105^, and is typically linked to water limited environments^106^ such as those from Mediterranean areas and other semi-arid regions.

Although reasons for greater reproductive success of Cabrera voles under genetic monogamy remain uncertain, this may be understood considering the sperm competition paradigm^107^. According to the sperm competition paradigm, whenever a female mates with more than one male, intra-sexual competition can occur in the reproductive track of females. Sperm competition hence describes the competition for fertilization between the sperm of more than one male for fertilization of females, and has been shown to favour an increase in sperm swimming velocity that maximizes the chances that the sperm will reach the ova before rival sperm^107^. While this sort of competition between males may be difficult to observe, it may have profound implications for male reproductive tactics in mammals^108^. In the case of the Cabrera vole, low levels of sperm competition have been suggested based on the sperm phenotypes linked to relatively small size, reduced morphological complexity and low swimming velocity^109^. In particular, the sperm of the Cabrera vole is relatively short, not very mobile and it lacks the hook in the acrosome, which is typically present is species with high sperm competition and multiple paternity^109^. This pattern supports the idea that Cabrera vole males have low sperm competition levels indicating limited ability to fertilize multiple females, thus investing reproductive efforts by guaranteeing regular access to a single breeding female, increasing paternity certainty and the chances of offspring survival. Low levels of sperm competition have been also suggested among other *Microtus* species adapted to limiting semi-arid Mediterranean environments from SW Europe (e.g. *M. duodecimcostatus* and *M. lusitanicus*)^109^, suggesting that such species could also be genetically monogamous^110^.

### Implications for conservation and research

Overall, our results indicated that the genetic monogamous mating system characteristic of Cabrera vole populations at low density also occurs in less common high density patches. This supports the idea that genetic monogamy in this species likely reflects a long-term evolutionary adaptation to the low productivity of Mediterranean habitats, rather than a facultative response to local population density. Therefore, ecologically, a locally high population density of Cabrera voles may essentially lead to intense competition for territories and nest sites, which could also favour investment in territory defence and mate guarding, potentially reducing, rather than increasing, the likelihood for extra-pair mating. This is supported by the relatively low estimates obtained for the spatial scale parameter *σ* in spatial density modelling, suggesting relatively smaller home-range sizes than those recorded in low-density patches^39,46^, with no significant differences between sexes. Therefore, in light of the apparent prevalence of genetic monogamy in the Cabrera vole across population density levels, we suggest that this threatened Iberian endemism may be more prone to lose genetic diversity compared to other species exhibiting social monogamy or able to switch to non-monogamous mating systems under certain socio-ecological contexts such as above-average density scenarios. Because multiple paternity tends to increase the effective population size and may help buffer the loss of genetic diversity associated to sudden demographic bottlenecks, genetically monogamous species may require particular attention when ranking conservation priorities for habitat management^21^. In this context, based on our findings, we suggest that maintaining large habitat patches is critical to provide breeding opportunities to several mating pairs and their offspring^111,112^. Habitat stability should be particularly relevant to improve opportunities for individuals to maintain their territories and mates, thus likely resulting in greater reproductive outputs^112^. This may be particularly relevant in those parts of the habitat less prone to dryness or flooding, where, according to our results, individuals tend to concentrate their activity. In parallel, conservation measures should also consider improving connectivity among habitat patches to allow unpaired individuals to disperse from high-density patches to other alternative habitat patches. This may be notably important in the case of females as these will define the upper limit to the number of males that can breed successfully. In this context, while our study was not designed to assess inter-patch movement by voles, it did revealed the occurrence of vole dispersal between the 2 studied habitat patches that were 1.8 km apart, as inferred by the occurrence of full siblings from 3 different family groups in both study sites. This dispersal distance is close to the median estimated for the species (around 2 km^113^), and together with the high vole density recorded, could be suggestive of a relatively favourable conservation status of Cabrera vole populations in the study region from Extremadura. However, further studies are needed to fully clarify this, as well as to evaluate whether other environmental factors (or interaction of factors) not considered here could potentially influence voles’ reproductive strategies and behaviours.

## Conclusion

The Cabrera vole represents an ancient anagenetic lineage within the *Microtus* genus (the *Iberomys*) that has maintained sufficient evolutionary flexibility to persist through substantial environmental change since the end of the early Pleistocene^44,114^, despite the recent declining population trends^115^. Our study, however, revealed that this flexibility should not extend to reproductive behaviours, as the species appears to exhibit limited plasticity in its genetically monogamous mating system and associated behaviours, regardless of population density. This may be related to the relatively low levels of sperm competition suggested for the species, with males improving their reproductive success by investing in establishing strong bonds to a single breeding female rather than attempting to copulate to multiple mates, even when chances for doing so appear to increase. On the other hand, paternal care (together with possible cooperative breeding by unpaired females) will also enhance the reproductive success of breeding females, thus resulting in mutual benefits for both sexes and increasing offspring survival. The predominance of genetic monogamy in the Cabrera vole, linked to a social organization indicative of high investment in offspring survival, is probably an adaptation to the dry Mediterranean environments, which are primarily shaped by aridity and water scarcity. Given the overall unfavourable trends of Cabrera vole population across the species distribution range mainly due to habitat destruction^56,116^, and that genetic monogamy is usually linked to lower effective population sizes and genetic diversity compared to extra-pair mating^117^, we suggest that the results from our study raise even further conservation concern relative to this threatened species, particularly considering the current trends in land use and climate change affecting the populations across the species’ distribution range^116,118,119^.

## Supplementary Information

Supplementary Information 1.

Supplementary Information 2.

## Data Availability

All data produced or analysed during this study are contained within this article and its Supplementary Information files. The genotyped microsatellite sequences are available in Genbank (accession numbers MH264524, MH264526, MH264528, MH264520, AB016154, EF666981, EF666983, EF666987, EF409379, EF177204, and MH264521). Codes used in density modelling are available at Zenodo repository: https://zenodo.org/records/14,470,066.

## References

[1] Shuster, S. M. & Wade, M. J. *Mating Systems and Strategies* (Princeton University Press, 2003).

[2] Emlen, S. T. & Oring, L. W. Ecology, sexual selection, and the evolution of mating systems. *Science* **197**, 215–223. 10.1126/science.327542 (1977). 327542 10.1126/science.327542

[3] Komers, P. E. & Brotherton, P. N. M. Female space use is the best predictor of monogamy in mammals. *Proc. R. Soc. Lond. B* **264**, 1261–1270. 10.1098/rspb.1997.0174 (1997). 10.1098/rspb.1997.0174 PMC1688588 9332011

[4] Kleiman, D. G. Monogamy in mammals. *Q. Rev. Biol.* **52**, 39–69. 10.1086/409721 (1977). 857268 10.1086/409721

[5] Klug, H. Why Monogamy? A review of potential ultimate drivers. *Front. Ecol. Evol.* **6**, 30. 10.3389/fevo.2018.00030 (2018).

[6] Carter, C. S. & Perkeybile, A. M. The monogamy paradox: What do love and sex have to do with it?. *Front. Ecol. Evol* **6**, 202. 10.3389/fevo.2018.00202 (2018). 31840025 10.3389/fevo.2018.00202 PMC6910656

[7] Kvarnemo, C. Why do some animals mate with one partner rather than many? A review of causes and consequences of monogamy. *Biol. Rev. Camb. Philos. Soc.* **93**, 1795–1812. 10.1111/brv.12421 (2018). 29687607 10.1111/brv.12421

[8] Lukas, D. & Clutton-Brock, T. H. The evolution of social monogamy in mammals. *Science* **341**, 526–530. 10.1126/science.1238677 (2013). 23896459 10.1126/science.1238677

[9] Oliveira, R. F., Taborsky, M. & Brockmann, H. J. *Alternative Reproductive Tactics: An Integrative Approach* (Cambridge University Press, 2008).

[10] Madrid, J. E., Parker, K. J. & Ophir, A. G. Variation, plasticity, and alternative mating tactics: Revisiting what we know about the socially monogamous prairie vole. *Adv. Study Behav.* **52**, 203–242. 10.1016/bs.asb.2020.02.001 (2020).

[11] Solomon, N. G., Keane, B., Knoch, L. R. & Hogan, P. J. Multiple paternity in socially monogamous prairie voles (*Microtus ochrogaster*). *Can. J. Zool.* **82**(10), 1667–1671. 10.1139/z04-142 (2004).

[12] Wolff, J. O. Social biology of rodents. *Integr. Zool.* **2**, 193–204. 10.1111/j.1749-4877.2007.00062.x (2007). 21396036 10.1111/j.1749-4877.2007.00062.x

[13] Rice, M. A., Wong, G. H. & Ophir, A. G. Impacts of spatial learning on male prairie vole mating tactics in seminatural field enclosures are context dependent. *Anim. Behav.* **191**, 57–73. 10.1016/j.anbehav.2022.06.011 (2022).

[14] Ophir, A. G. Navigating monogamy: nonapeptide sensitivity in a memory neural circuit may shape social behavior and mating decisions. *Front. Neurosci.* **11**, 397. 10.3389/fnins.2017.00397 (2017). 28744194 10.3389/fnins.2017.00397 PMC5504236

[15] López-Gutiérrez, M. F., Mejía-Chávez, S., Alcauter, S. & Portillo, W. The neural circuits of monogamous behavior. *Front. Neural Circuits* **16**, 978344. 10.3389/fncir.2022.978344 (2022). 36247729 10.3389/fncir.2022.978344 PMC9559370

[16] Huck, M., Fernandez-Duque, E., Babb, P. & Schurr, T. Correlates of genetic monogamy in socially monogamous mammals: insights from Azara’s owl monkeys. *Proc. R. Soc. Lond. Biol. Sci.* **281**, 20140195. 10.1098/rspb.2014.0195 (2014). 10.1098/rspb.2014.0195 PMC3973279 24648230

[17] Lambert, C. T., Sabol, A. C. & Solomon, N. G. Genetic monogamy in socially monogamous mammals is primarily predicted by multiple life history factors: A meta-analysis. *Front. Ecol. Evol.* **6**, 139. 10.3389/fevo.2018.00139 (2018).

[18] Lee, A. M., Sather, B. E. & Engen, S. Demographic stochasticity, Allee effects, and extinction: The influence of mating system and sex ratio. *Am. Nat.* **177**, 301–313. 10.1086/658344 (2011). 21460539 10.1086/658344

[19] Leach, D., Ak, S. & Weiss-Lehman, C. Stochasticity in social structure and mating system drive extinction risk. *Ecosphere* **11**(2), e03038. 10.1002/ecs2.3038 (2020).

[20] Morino, L. Monogamy in mammals: expanding the perspective on Hylobatid mating systems. In *The gibbons: new perspectives on small ape socioecology and population biology (Lappan, S* (ed. Whittaker, D. J.) 279–312 (Springer, 2009).

[21] Dudash, M. R., & Murren, C. J. The influence of breeding systems and mating systems on conservation genetics and conservation decisions. In *Conservation Biology: Evolution in Action* (eds. Carroll, S. P. & Fox, C. W.) 68–80 (Oxford, UK: Oxford University Press. 2008).

[22] Shuster, S. & Wade, M. Equal mating success among male reproductive strategies in a marine isopod. *Nature* **350**, 608–610. 10.1038/350608a0 (1991).

[23] Clutton-Brock, T. Social evolution in mammals. *Science* **373**, eabc9699. 10.1126/science.abc9699 (2021). 34529471 10.1126/science.abc9699

[24] Thompson, W. *Sampling Rare or Elusive Species: Concepts, Designs, and Techniques for Estimating Population Parameters*. (Island Press. 2013)

[25] Ferreira, C. M. et al. Genetic non-invasive sampling (gNIS) as a cost-effective tool for monitoring elusive small mammals. *Eur. J. Wildl. Res.* **64**, 46. 10.1007/s10344-018-1188-8 (2018).

[26] Proença-Ferreira, A. et al. Drivers of survival in a small mammal of conservation concern: an assessment using extensive genetic non-invasive sampling in fragmented farmland. *Biol. Conserv.* **230**, 131–140. 10.1016/j.biocon.2018.12.021 (2019).

[27] Cohas, A. & Allainé, D. Social structure influences extra-pair paternity in socially monogamous mammals. *Biol. Lett.* **5**, 313–316. 10.1098/rsbl.2008.0760 (2009). 19324647 10.1098/rsbl.2008.0760 PMC2679913

[28] Dobson, F. S., Way, B. M. & Baudoin, C. Spatial dynamics and the evolution of social monogamy in mammals. *Behav. Ecol.* **21**, 747–752. 10.1093/beheco/arq048 (2010).

[29] Streatfeild, C. A., Mabry, K. E., Keane, B., Crist, T. O. & Solomon, N. G. Intraspecific variability in the social and genetic mating systems of prairie voles. *Microtus ochrogaster. Anim. Behav.* **82**(6), 1387–1398. 10.1016/j.anbehav.2011.09.023 (2011).

[30] Sabol, A. C., Lambert, C. T., Keane, B., Solomon, N. G. & Dantzer, B. How does individual variation in sociality influence fitness in prairie voles?. *Anim. Behav.* **163**, 39–49. 10.1016/j.anbehav.2020.02.009 (2020).

[31] Forero, S. A. & Ophir, A. G. Multi-level effects driving cognitive and behavioral variability among prairie voles: Insights into reproductive decision-making from biological levels of organization. *Brain Behav. Evol.* **97**, 225–240. 10.1159/000522109 (2022). 35051922 10.1159/000522109 PMC9256755

[32] Ophir, A. G., Phelps, S. M., Sorin, A. B. & Wolff, J. O. Social but not genetic monogamy is associated with greater breeding success in prairie voles. *Anim. Behav.* **75**, 1143–1154. 10.1016/j.anbehav.2007.09.022 (2008).

[33] Busher, P. Social organization and monogamy in the beaver. In *Rodent societies: An ecological and ecolutionary perspective* (eds. Wolff, J. O. & Sherman, P. W.) 280–290 (Chicago, IL: University of Chicago Press. 2007).

[34] Endries, M. J. & Adler, G. H. Spacing patterns of a tropical forest rodent, the spiny rat (Proechimys semispinosus), in Panama. *J. Zool.* **265**, 147–155. 10.1017/S0952836904006144 (2005).

[35] Asher, M., de Oliveira, E. S. & Sachser, N. Social system and spatial organization in wild guinea pigs (*Cavia aperea*) in a natural population. *J. Mammal.* **85**, 788–796. 10.1644/BNS-012 (2004).

[36] Natoli, E. et al. Genetic inference of the mating system of free-ranging domestic dogs. *Behav. Ecol.* **32**(4), 646–656. 10.1093/beheco/arab011 (2021). 34539241 10.1093/beheco/arab011 PMC8444980

[37] Plesnar-Bielak, A., Skrzynecka, A. M., Prokop, Z. M. & Radwan, J. Mating system affects population performance and extinction risk under environmental challenge. *Proc. R. Soc. B* **279**, 4661–4667. 10.1098/rspb.2012.1867 (2012). 22977151 10.1098/rspb.2012.1867 PMC3479737

[38] Fernández-Salvador, R., García-Perea, R. & Ventura, J. Reproduction and postnatal growth of the Cabrera vole, Microtus cabrerae, in captivity. *Can. J. Zool.* **79**, 2080–2085. 10.1139/cjz-79-11-2080 (2001).

[39] Pita, R., Mira, A. & Beja, P. Spatial segregation of two vole species (*Arvicola sapidus* and *Microtus cabrerae*) within habitat patches in a highly fragmented farmland landscape. *Eur. J. Wildl. Res.* **56**, 651–662. 10.1007/s10344-009-0360-6 (2010).

[40] Gomes, L., Salgado, P., Barata, E. N. & Mira, A. The effect of pair bonding in Cabrera vole’s scent marking. *Acta Ethol.* **16**, 181–188. 10.1007/s10211-013-0151-7 (2013).

[41] Lópes-Fernandes, M., Pita, R., Mira, A. *Microtus cabrerae*. The IUCN Red List of Threatened Species 2019: E.T13418A90931498. https://www.iucnredlist.org/species/13418/90931498 (2019).

[42] Luque-Larena, J. J. & López, P. Microhabitat use by wild-ranging Cabrera voles Microtus cabrerae as revealed by live trapping. *Eur. J. Wildl. Res.* **53**, 221–225. 10.1007/s10344-006-0084-9 (2007).

[43] Pita, R., Mira, A. & Beja, P. Assessing habitat differentiation between coexisting species: The role of spatial scale. *Acta Oecol.* **37**, 124–132. 10.1016/j.actao.2011.01.006 (2011).

[44] Pita, R., Mira, A. & Beja, P. Microtus cabrerae (Rodentia: Cricetidae). *Mamm. Species* **46**(912), 48–70. 10.1644/912.1 (2014).

[45] Rosário, I. T. Towards a conservation strategy for an endangered rodent, the Cabrera vole (*Microtus cabrerae* Thomas). Insights from ecological data. Ph.D. dissertation (University of Lisbon, Lisbon, Portugal, 2012).

[46] Sabino-Marques, H. et al. Combining genetic non-invasive sampling with spatially explicit capture-recapture models for density estimation of a patchily distributed small mammal. *Eur. J. Wildl. Res.* **64**, 44. 10.1007/s10344-018-1206-x (2018).

[47] Peralta, D. et al. From species detection to population size indexing: The use of sign surveys for monitoring a rare and otherwise elusive small mammal. *Eur. J. Wildl. Res.* **69**, 9. 10.1007/s10344-022-01634-2 (2023).

[48] Fernández-Salvador, R., Ventura, J. & García-Perea, R. Breeding patterns and demography of a population of the Cabrera vole, Microtus cabrerae. *Anim. Biol.* **55**, 147–161. 10.1163/1570756053993497 (2005).

[49] Ventura, J., López-Fuster, M. J. & Cabrera-Mllet, M. The Cabrera vole, *Microtus cabrerae*, in Spain: A biological and a morphometric approach. *Neth. J. Zool.* **48**, 83–100. 10.1163/156854298X00237 (1998).

[50] Fernández-Salvador, R., García-Perea, R. & Ventura, J. Effect of climatic fluctuations on body mass of a Mediterranean vole, Microtus cabrerae. *Mammal. Biol.* **70**(2), 73–83. 10.1016/j.mambio.2004.06.002 (2005).

[51] Landete-Castillejos, T., Andres-Abellan, M., Argandona, J. J. & Garde, J. Distribution of the Cabrerae vole (*Microtus cabrerae*) in its first reported areas reassessed by live-trapping. *Biol. Conserv.* **94**, 127–130. 10.1016/S0006-3207(99)00167-6 (2000).

[52] Mougeot, F., Lambin, X., Rodriguez-Pastor, R. & RomaironeLuque-Larena, J. J. J. Numerical response of a mammalian specialist predator to multiple prey dynamics in Mediterranean farmlands. *Ecology* **100**(9), e02776. 10.1002/ecy.2776 (2019). 31172505 10.1002/ecy.2776

[53] Andreassen, H. P. et al. Population cycles and outbreaks of small rodents: Ten essential questions we still need to solve. *Oecologia* **195**, 601–622. 10.1007/s00442-020-04810-w (2021). 33369695 10.1007/s00442-020-04810-w PMC7940343

[54] De León Llamazares, A. (1991). Caracterización Agroclimática de la Provincia de Cáceres. (Ministerio de Agricultura, Pesca y Alimentación, Madrid, 1991).

[55] Garrido-García, J. A. & Soriguer, R. C. Topillo de Cabrera *Iberomys cabrerae* (Thomas, 1906). Guía de indícios de los mamíferos de España. SECEM. http://www.secem.es/wp-content/uploads/2015/07/020-Iberomys-cabrerae.pdf (2015).

[56] Palomo, L. J., Gisbert, J. & Blanco, J. C. Atlas y libro rojo de los mamíferos terrestres de España. (Dirección General para la Biodiversidad, SECEM, SECEMU, Madrid. 2007)

[57] Maudet, C., Luikart, G., Dubray, D., Von Hardenberg, A. & Taberlet, P. Low genotyping error rates in wild ungulate faeces sampled in winter. *Mol. Ecol. Notes* **4**(4), 772–775. 10.1111/j.1471-8286.2004.00787.x (2004).

[58] Ishibashi, Y. et al. Polymorphic microsatellite DNA markers in the field vole *Microtus montebelli*. *Mol. Ecol.* **8**, 163–164. 10.3106/mammalstudy.22.5 (1999). 9919707

[59] Walser, B. & Heckel, G. Microsatellite markers for the common vole (*Microtus arvalis*) and their cross-species utility. *Conserv. Genet.* **9**, 479–481. 10.1007/s10592-007-9355-6 (2007).

[60] Jaarola, M., Ratkiewicz, M., Ashford, R. T., Brunhoff, C. & Borkowska, A. Isolation and characterization of polymorphic microsatellite loci in the field vole, *Microtus agrestis*, and their cross-utility in the common vole, Microtus arvalis. *Mol. Ecol. Notes* **7**, 1029–1031. 10.1111/j.1471-8286.2007.01763.x (2007).

[61] Gauffre, B., Galan, M., Bretagnolle, V. & Cosson, J. F. Polymorphic microsatellite loci and PCR multiplexing in the common vole, Microtus arvalis. *Mol. Ecol. Notes* **7**, 830–832. 10.1111/j.1471-8286.2007.01718.x (2007).

[62] Alasaad, S. et al. Applicability of mitochondrial DNA for the identification of Arvicolid species from faecal samples: A case study from the threatened Cabrera’s vole. *Mol. Ecol. Res.* **11**, 409–414. 10.1111/j.1755-0998.2010.02939.x (2011). 10.1111/j.1755-0998.2010.02939.x 21429155

[63] Beja-Pereira, A., Oliveira, R., Alves, P. C., Schwartz, M. K. & Luikart, G. Advancing ecological understandings through technological transformations in noninvasive genetics. *Mol. Ecol. Res.* **9**, 1279–1301. 10.1111/j.1755-0998.2009.02699.x (2009). 10.1111/j.1755-0998.2009.02699.x 21564900

[64] Barbosa, S., Paupério, J., Searle, J. B. & Alves, P. C. Genetic identification of Iberian rodent species using both mitochondrial and nuclear loci: Application to noninvasive sampling. *Mol. Ecol. Res.* **13**, 43–56. 10.1111/1755-0998.12024 (2013). 10.1111/1755-0998.12024 23095787

[65] Costa, V., Rosenbom, S., Monteiro, R., O’Rourke, S. M. & Beja-Pereira, A. Improving DNA quality extracted from fecal samples - a method to improve DNA yield. *Eur. J. Wildl. Res.* **63**, 3. 10.1007/s10344-016-1058-1 (2017).

[66] Kearse, M. et al. Geneious Basic: An integrated and extendable desktop software platform for the organization and analysis of sequence data. *Bioinformatics* **28**, 1647–1649. 10.1093/bioinformatics/bts199 (2012). 22543367 10.1093/bioinformatics/bts199 PMC3371832

[67] Valière, N. GIMLET: A computer program for analysing genetic individual identification data. *Mol. Ecol. Notes* **2**, 377–379. 10.1046/j.1471-8286.2002.00228.x-i2 (2002).

[68] Johnson, P. C. D. & Haydon, D. T. Maximum-likelihood estimation of allelic dropout and false allele error rates from microsatellite genotypes in the absence of reference data. *Genetics* **175**, 827–842. 10.1534/genetics.106.064618 (2007). 17179070 10.1534/genetics.106.064618 PMC1800638

[69] Lombart, T. adegenet: A R package for the multivariate analysis of genetic markers. *Bioinformatics* **24**, 1403–1405. 10.1093/bioinformatics/btn129 (2008). 18397895 10.1093/bioinformatics/btn129

[70] Paradis, E. pegas: An R package for population genetics with an integrated–modular approach. *Bioinformatics* **26**, 419–420. 10.1093/bioinformatics/btp696 (2010). 20080509 10.1093/bioinformatics/btp696

[71] R Core Team R: A Language and Environment for Statistical Computing_. R Foundation for Statistical Computing, Vienna, Austria. https://www.R-project.org/ (2023).

[72] Efford, M. G. Density estimation in live-trapping studies. *Oikos* **106**(3), 598–610. 10.1111/j.0030-1299.2004.13043.x (2004).

[73] Royle, J. A. & Young, K. V. A hierarchical model for spatial capture–recapture data. *Ecology* **89**(8), 2281–2289. 10.1890/07-0601.1 (2008). 18724738 10.1890/07-0601.1

[74] Romairone, J., Jimenez, J., Luque-Larena, J. J. & Mougeot, F. Spatial capture-recapture design and modelling for the study of small mammals. *PLoS ONE* **13**(6), e0198766. 10.1371/journal.pone.0198766 (2018). 29879211 10.1371/journal.pone.0198766 PMC5991742

[75] Borchers, D. L. & Efford, M. G. Spatially explicit maximum likelihood methods for capture–recapture studies. *Biometrics* **64**(2), 377–385. 10.1111/j.1541-0420.2007.00927.x (2008). 17970815 10.1111/j.1541-0420.2007.00927.x

[76] Efford, M. G. & Fewster, R. M. Estimating population size by spatially explicit capture–recapture. *Oikos* **122**, 918–928. 10.1111/j.1600-0706.2012.20440.x (2013).

[77] Royle, J. A., Chandler, R. B., Sollmann, R. & Gardner, B. *Spatial Capture-Recapture* (Academic Press, Waltham, Massachusetts, 2013).

[78] Durbach, I. et al. That’s not the Mona Lisa! How to interpret spatial capture-recapture density surface estimates. *Biometrics* **80**(1), 1–9. 10.1093/biomtc/ujad020 (2024). 10.1093/biomtc/ujad020 38364802

[79] López-Bao, J. V. et al. Consistent bear population DNA-based estimates regardless molecular markers type. *Biol. Conserv.* **248**, 108651. 10.1016/j.biocon.2020.108651 (2020).

[80] Milleret, C. et al. Using partial aggregation in spatial capture recapture. *Methods Ecol. Evol.* **9**, 1896–1907. 10.1111/2041-210X.13030 (2018).

[81] Royle, J. A. & Converse, S. J. Hierarchical spatial capture-recapture models: Modelling population density in stratified populations. *Methods Ecol. Evol.* **5**, 37–43. 10.1111/2041-210X.12135 (2014).

[82] Da Rocha, D. G., Sollmann, R., Ramalho, E. E., Ilha, R. & Tan, C. K. W. Ocelot (*Leopardus pardalis*) density in Central Amazonia. *PLoS ONE* **11**(5), e0154624. 10.1371/journal.pone.0154624 (2016). 27191598 10.1371/journal.pone.0154624 PMC4871438

[83] Gelin, M. L. et al. Response of pumas (*Puma concolor*) to migration of their primary prey in Patagonia. *PLoS ONE* **12**(12), e0188877. 10.1371/journal.pone.0188877 (2017). 29211753 10.1371/journal.pone.0188877 PMC5718558

[84] Morin, D. J., Waits, L. P., McNitt, D. C. & Kelly, M. J. Efficient single-survey estimation of carnivore density using fecal DNA and spatial capture-recapture: A bobcat case study. *Popul. Ecol.* **60**, 197–209. 10.1007/s10144-018-0606-9 (2018).

[85] Jiménez, J. et al. Restoring apex predators can reduce mesopredator abundances. *Biol. Conserv.* **238**, 108234. 10.1016/j.biocon.2019.108234 (2019).

[86] Royle, J. A., Dorazio, R. M. & Link, W. A. Analysis of multinomial models with unknown index using data augmentation. *J. Comput. Graph. Stat.* **16**, 67–85. 10.1198/106186007X181425 (2007).

[87] Jiménez, J., Augustine, B. C., Linden, D. W., Chandler, R. B. & Royle, J. A. Spatial capture-recapture with random thinning for unidentified encounters. *Ecol. Evol.* **11**, 1187–1198. 10.1002/ece3.7091 (2021). 33598123 10.1002/ece3.7091 PMC7863675

[88] Meredith, M. *makeJAGSmask: Construct a Habitat Matrix for Use with SECR Analysis in JAGS or BUGS*. R package version 0.1.1.9005. https://rdrr.io/github/mikemeredith/makeJAGSmask/ (2021).

[89] NIMBLE Development Team “nimbleSMC: Sequential Monte Carlo methods for NIMBLE.” 10.5281/zenodo.1211190 (2024).

[90] Gelman, A. et al. *Bayesian Data Analysis* (3rd ed.) Vol. 3. (CRC Press. 2013).

[91] Plummer, M., Best, N., Cowles, K. & Vines, K. CODA: Convergence diagnosis and output analysis for MCMC. *R News* **6**, 7–11 (2006).

[92] Jones, O. R. & Wang, J. COLONY: A program for parentage and sibship inference from multilocus genotype data. *Mol. Ecol. Resour.* **10**, 551–555. 10.1111/j.1755-0998.2009.02787.x (2010). 21565056 10.1111/j.1755-0998.2009.02787.x

[93] Reardon, S. F. & O’Sullivan, D. Measures of spatial segregation. *Sociol. Methodol.* **34**, 121–162. 10.1111/j.0081-1750.2004.00150.x (2004).

[94] Hong, S.-Y., O’Sullivan, D. & Sadahiro, Y. Implementing spatial segregation measures in R. *PLoS ONE* **9**(11), e113767. 10.1371/journal.pone.0113767 (2014). 25415326 10.1371/journal.pone.0113767 PMC4240605

[95] Dolotovskaya, S., Roos, C. & Heymann, E. W. Genetic monogamy and mate choice in a pair-living primate. *Sci. Rep.* **10**, 20328. 10.1038/s41598-020-77132-9 (2020). 33230212 10.1038/s41598-020-77132-9 PMC7683532

[96] Corley, M. et al. Inbreeding avoidance, competition and natal dispersal in a pair-living, genetically monogamous mammal, Azara’s owl monkey (Aotus azarae). *R. Soc. Open Sci.* **11**, 240379. 10.1098/rsos.240379 (2024). 39113772 10.1098/rsos.240379 PMC11305132

[97] Chesser, R. K. Influence of gene flow and breeding tactics on gene diversity within populations. *Genetics* **129**, 573–583. 10.1093/genetics/129.2.573 (1991). 1743493 10.1093/genetics/129.2.573 PMC1204645

[98] Biagolini, C. Jr., Westneat, D. F. & Francisco, M. R. Does habitat structural complexity influence the frequency of extra-pair paternity in birds?. *Behav. Ecol. Sociobiol.* **71**, 101. 10.1007/s00265-017-2329-x (2017).

[99] Erhardt, S., Förschler, M. I. & Fietz, J. Is promiscuity the key? Multiple paternity in the garden dormouse (*Eliomys quercinus*) *Mamm*. *Biol.* **104**, 395–405. 10.1007/s42991-024-00414-6 (2024).

[100] Westneat, D. F. & Gray, E. M. Breeding synchrony and extrapair fertilizations in two populations of Red-winged Blackbirds. *Behav. Ecol.* **9**, 456–464 (1998).

[101] Opie, C., Atkinson, Q. D., Dunbar, R. I. M. & Shultz, S. Male infanticide leads to social monogamy in primates. *P. Natl. Acad. Sci. USA* **110**, 13328–13332. 10.1073/pnas.1307903110 (2013). 10.1073/pnas.1307903110 PMC3746880 23898180

[102] Ribble, D. O. The evolution of social and reproductive monogamy in Peromyscus: Evidence from *Peromyscus californicus* (the California mouse). In *Monogamy: Mating strategies and partnerships in birds, humans and other mammals* (eds. Reichard, U. H. & Boesch, C.) pp. 81–92 (Cambridge University Press. 2003) 10.1017/CBO9781139087247.005

[103] Smith, J., Jaeggi, A., Holmes, R. & Silk, J. Sex differences in cooperative coalitions: A mammalian perspective. *Philos. Trans. R. Soc. B: Biol. Sci.* **378**, 20210426. 10.1098/rstb.2021.0426 (2022). 10.1098/rstb.2021.0426 PMC9703251 36440559

[104] Rousset, F. & Billiard, S. A theoretical basis for measures of kin selection in subdivided populations: Finite populations and localized dispersal. *J. Evol. Biol.* **13**, 814–825. 10.1046/j.1420-9101.2000.00219.x (2000).

[105] Lukas, D. & Clutton-Brock, T. Cooperative breeding and monogamy in mammalian societies. *Proc. R. Soc. Lond. B* **279**, 2151–2156. 10.1098/rspb.2011.2468 (2012). 10.1098/rspb.2011.2468 PMC3321711 22279167

[106] Waterman, J. Male mating strategies in rodents. In *Rodent societies: An ecological and evolutionary perspective* (eds. Wolff, J. O. & Sherman, P. W.) 27–41 (The University of Chicago Press. Chicago, IL, USA. 2007).

[107] Parker, G. A. Sperm competition and its evolutionary consequences in insects. *Biol. Rev. Camb. Philos. Soc.* **45**, 525–567. 10.1111/j.1469-185X.1970.tb01176.x (1970).

[108] Gomendio, M. & Roldan, E. R. S. Sperm competition influences sperm size in mammals. *Proc. R. Soc. Lond. B BioI. Sci.* **243**, 181–185. 10.1098/rspb.1991.0029 (1991). 10.1098/rspb.1991.0029 1675796

[109] Montoto, L. G. et al. Sperm competition differentially affects swimming velocity and size of spermatozoa from closely related muroid rodents: Head first. *Reproduction* **142**(6), 819–830. 10.1530/REP-11-0245 (2011). 21954130 10.1530/REP-11-0232

[110] Ventura, J., Gisbert, J. & Jiménez, L. Breeding characteristics of the Lusitanian pine vole, Microtus lusitanicus. *Anim. Biol.* **60**(1), 1–14. 10.1163/157075610X12610595764011 (2010).

[111] Pita, R., Beja, P. & Mira, A. Spatial population structure of the Cabrera vole in Mediterranean farmland: The relative role of patch and matrix effects. *Biol. Conserv.* **134**, 383–392. 10.1016/j.biocon.2006.08.026 (2007).

[112] Crispim-Mendes, T. et al. Patch spatial attributes and time to disturbance affect the emergence of source local populations within ephemeral habitats. *Ecol. Model.* **496**, 110830. 10.1016/j.ecolmodel.2024.110839 (2024).

[113] Mestre, F., Risk, B. B., Mira, A., Beja, P. & Pita, R. A metapopulation approach to predict species range shifts under different climate change and landscape connectivity scenarios. *Ecol. Model.* **359**, 406–414. 10.1016/j.ecolmodel.2017.06.013 (2017).

[114] Barbosa, S. et al. Endemic species may have complex histories: within refugium phylogeography of an endangered Iberian vole. *Mol. Ecol.* **26**, 951–967. 10.1111/mec.13994 (2017). 28028865 10.1111/mec.13994

[115] Mestre, F. et al. Inferring past refugia and range dynamics through the integration of fossil, niche modelling and genomic data. *J. Biogeogr.* **49**, 2064–2076. 10.1111/jbi.14492 (2022).

[116] Barbosa, S. Mestre, F., Pita, R. *Microtus cabrerae* rato-de-Cabrera. In *Livro Vermelho dos Mamíferos de Portugal Continental*. (eds. Mathias M. L. et al.) 152–153 (FCiências.ID, ICNF, Lisboa. 2023)

[117] Rafajlovic, M. et al. The effect of multiple paternity on genetic diversity of small populations during and after colonisation. *PLoS ONE* **8**, e75587. 10.1371/journal.pone.0075587 (2013). 24204577 10.1371/journal.pone.0075587 PMC3810386

[118] Mestre, F. et al. Combining distribution modelling and non-invasive genetics to improve range shift forecasting. *Ecol. Model.* **297**, 171–179. 10.1016/j.ecolmodel.2014.11.018 (2015).

[119] Garrido-García, J. A., Nieto-Lugilde, D., Alba-Sánchez, F. & Soriguer, R. C. Agricultural intensification during the Late Holocene rather than climatic aridification drives the population dynamics and the current conservation status of *Microtus cabrerae*, an endangered Mediterranean rodent. *J. Biogeogr.* **45**, 448–460. 10.1111/jbi.13134 (2018).

