# Supplementary material for: Genetic monogamy in high density populations of a threatened Mediterranean rodent
Source: Sci Rep. 2025 Jul 4;15:23840. doi: 10.1038/s41598-025-09003-0 (PMC12229526; doi:10.1038/s41598-025-09003-0)
Supplement: Supplementary file 2 — Supplementary Information 2. [file 41598_2025_9003_MOESM2_ESM.pdf]

SUPPLEMENTARY INFORMATION

Genetic monogamy in high density populations of a threatened Mediterranean rodent

Ricardo Pita, José Jiménez, Joana Paupério, Benigno Cienfuegos, Alejandro Chamizo de Castro, Joshua Díaz-Caballero, João Queirós, Clara Ferreira, François Mougeot, Alfredo Anega, Maria Jesús Palacios, Juan José Luque-Larena

Table S1 – Summary of genotyping error rates of the 10 microsatellite loci used for genotyping Cabrera vole faecal samples collected from the two study sites in Extremadura

		Replicate						Average	%
		**01/fev**	**01/mar**	**01/abr**	**02/mar**	**02/abr**	**03/abr**		
**Mc18**	Dropout	0	0	0.000001	0.000004	0	0.000001	0	0.00%
	False Allele	0	0	0	0	0.000001	0	0	0.00%
**Mc24**	Dropout	0	0.000001	0.000001	0.000001	0.000001	0.000001	0	0.00%
	False Allele	0	0	0	0	0	0	0	0.00%
**Mc30**	Dropout	0.000001	0.01278	0	0.01278	0	0.01278	0.00639	0.64%
	False Allele	0	0	0	0	0	0	0	0.00%
**Ma25**	Dropout	0.008217	0.01032	0.008287	0.00619	0.008289	0.010411	0.00862	0.86%
	False Allele	0	0	0	0	0	0	0	0.00%
**MAG25**	Dropout	0.000001	0.001886	0	0.000002	0.000001	0.001886	0.00063	0.06%
	False Allele	0	0	0.001646	0	0	0	0.00027	0.03%
**mar/76**	Dropout	0.050227	0.05275	0.068904	0.085912	0.081001	0.091282	0.07168	7.17%
	False Allele	0.006118	0.003055	0	0.003497	0.003406	0.003335	0.00324	0.32%
**Mc02**	Dropout	0.002487	0.002489	0.000001	0.000001	0.002489	0.002489	0.00166	0.17%
	False Allele	0	0	0	0	0	0	0	0.00%
**MSMM-3**	Dropout	0	0.000001	0.000001	0.000001	0	0.000001	0	0.00%
	False Allele	0	0	0.000001	0.000001	0.000001	0.000001	0	0.00%
**mar/03**	Dropout	0.003965	0.003964	0.003965	0	0	0	0.00198	0.20%
	False Allele	0	0.000004	0	0.000001	0	0	0	0.00%
**mar/16**	Dropout	0.002075	0.000001	0.000001	0.002076	0.002076	0	0.00104	0.10%
	False Allele	0	0	0	0.000001	0	0	0	0.00%

Table S2 – Summary characteristics of the 10 microsatellite loci used for genotyping Cabrera vole faecal samples collected from the two study sites in Extremadura Na, number of alleles; HO, observed heterozygosity; HE, expected heterozygosity; FIS, inbreeding coefficient.

Locus	Na	HO	HE	FIS
Mc18	10	0.82	0.78	-0.053
Mc24	5	0.37	0.38	0.031
Mc30	9	0.14	0.42	0.675
Ma25	12	0.82	0.85	0.038
MAG25	8	0.88	0.82	-0.068
Mar76	8	0.59	0.6	0.016
Mc02	5	0.69	0.71	0.04
MSMM3	8	0.76	0.77	0.019
Mar03	6	0.84	0.8	-0.049
Mar16	6	0.79	0.8	-0.005
**Total**	77	0.67	0.69	0.064

Table S3 – Identification accuracy of full-sibs in COLONY under genetic monogamy, promiscuity, polygyny, and polyandry mating systems. No half-sibs were detected for any of the non-monogamous mating systems considered.

MONOGAMY

Type	Dyad1	Dyad2	Site
Full-sib	1-F-1	118-F-1	1
Full-sib	1-F-1	119-F-1	1
Full-sib	1-F-1	8-M-1	1
Full-sib	101-F-1	133-M-1	1
Full-sib	101-F-1	77-M-1	1
Full-sib	107-F-2	108-M-2	2
Full-sib	118-F-1	119-F-1	1
Full-sib	119-F-1	8-M-1	1
Full-sib	126-F-1	40-M-2	1/2
Full-sib	127-F-1	134-F-1	1
Full-sib	132-F-1	22-M-1	1
Full-sib	14-M-2	113-M-2	2
Full-sib	16-F-1	71-F-1	1
Full-sib	2-F-1	19-F-1	1
Full-sib	21-M-1	22-M-1	1
Full-sib	23-F-2	24-M-2	2
Full-sib	26-M-2	39-M-2	2
Full-sib	35-F-2	28-M-2	2
Full-sib	4-F-1	20-F-2	1/2
Full-sib	54-F-2	55-F-2	2
Full-sib	67-F-1	120-M-1	1
Full-sib	67-F-1	68-F-1	1
Full-sib	68-F-1	120-M-1	1
Full-sib	71-F-1	96-M-1	1
Full-sib	77-M-1	133-M-1	1
Full-sib	8-M-1	117-M-1	1
Full-sib	84-F-2	58-M-2	2
Full-sib	85-F-2	115-F-2	2
Full-sib	88-M-2	104-M-2	2
Full-sib	89-F-2	81-M-2	2
Full-sib	89-F-2	98-M-1	1/2
Full-sib	9-F-1	67-F-1	1
Full-sib	9-F-1	68-F-1	1
Full-sib	90-F-1	117-M-1	1
Full-sib	90-F-1	118-F-1	1
Full-sib	95-F-1	128-M-1	1
Full-sib	99-F-1	12-M-1	1

PROMISCUITY

Type	Dyad1	Dyad2	Site
Full-sib	1-F-1	119-F-1	1
Full-sib	101-F-1	133-M-1	1
Full-sib	21-M-1	22-M-1	1
Full-sib	67-F-1	68-F-1	1
Full-sib	71-F-1	96-M-1	1
Full-sib	9-F-1	68-F-1	1

POLYGYNY

Type	Dyad1	Dyad2	Site
Maternity	19-F-1	10-F-1	1
Maternity	19-F-1	4-F-1	1
Full-sib	1-F-1	119-F-1	1
Full-sib	101-F-1	133-M-1	1
Full-sib	21-M-1	22-M-1	1
Full-sib	26-M-2	39-M-2	2
Full-sib	4-F-1	10-F-1	1
Full-sib	67-F-1	68-F-1	1
Full-sib	71-F-1	96-M-1	1
Full-sib	9-F-1	68-F-1	1

POLYANDRY

Type	Dyad1	Dyad2	Site
Full-sib	1-F-1	119-F-1	1
Full-sib	101-F-1	133-M-1	1
Full-sib	21-M-1	22-M-1	1
Full-sib	26-M-2	39-M-2	2
Full-sib	67-F-1	68-F-1	1
Full-sib	71-F-1	96-M-1	1
Full-sib	89-F-2	81-M-2	2
Full-sib	9-F-1	68-F-1	1

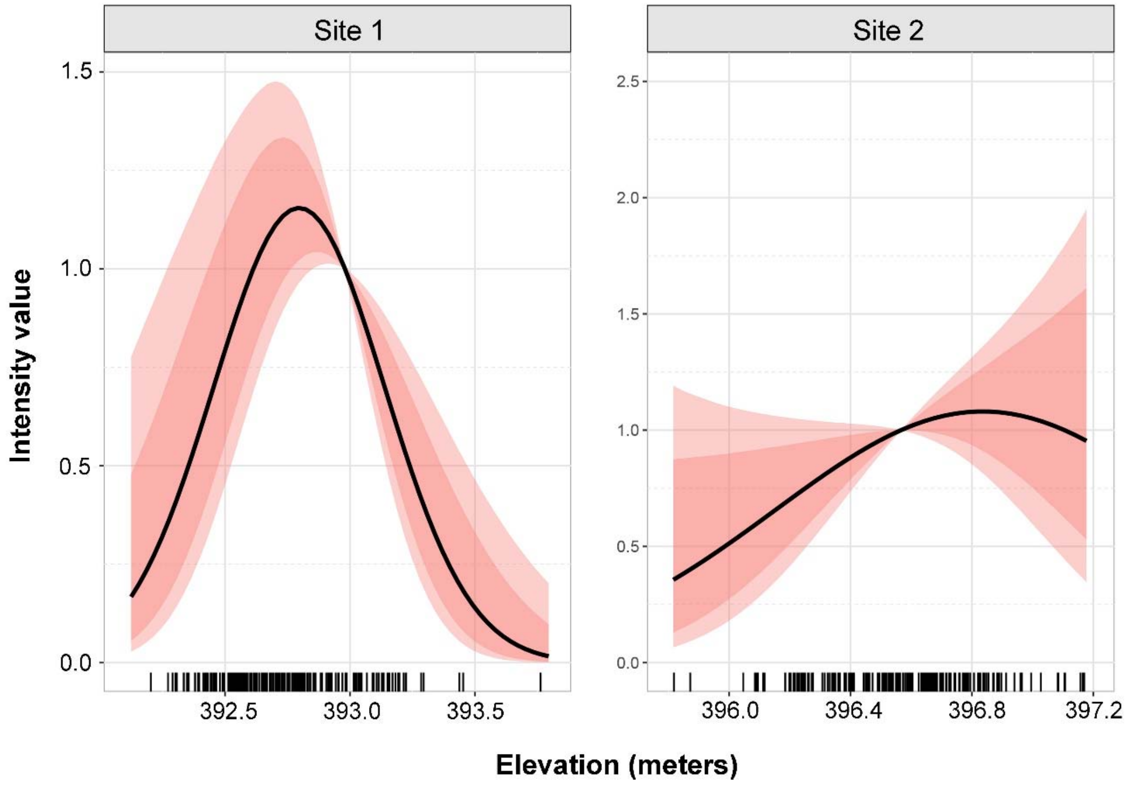

Figure S1 – Relationship between intensity function value of Cabrera voles' activity centres and fine scale variation in altitude within each study site as revealed by SCR.
